# “Hey Siri, Help Me Take Care of My Child”: A Feasibility Study With Caregivers of Children With Special Healthcare Needs Using Voice Interaction and Automatic Speech Recognition in Remote Care Management

**DOI:** 10.3389/fpubh.2022.849322

**Published:** 2022-03-03

**Authors:** Emre Sezgin, Brannon Oiler, Brandon Abbott, Garey Noritz, Yungui Huang

**Affiliations:** ^1^Information Technology Research and Innovation, The Abigail Wexner Research Institute, Nationwide Children's Hospital, Columbus, OH, United States; ^2^Department of Pediatrics, Nationwide Children's Hospital, Columbus, OH, United States

**Keywords:** voice interaction, mobile app, automatic speech recognition, children with special healthcare needs, voice assistant, remote care management, feasibility, patient-generated health data (PGHD)

## Abstract

**Background:**

About 23% of households in the United States have at least one child who has special healthcare needs. As most care activities occur at home, there is often a disconnect and lack of communication between families, home care nurses, and healthcare providers. Digital health technologies may help bridge this gap.

**Objective:**

We conducted a pre-post study with a voice-enabled medical note taking (diary) app (SpeakHealth) in a real world setting with caregivers (parents, family members) of children with special healthcare needs (CSHCN) to understand feasibility of voice interaction and automatic speech recognition (ASR) for medical note taking at home.

**Methods:**

In total, 41 parents of CSHCN were recruited. Participants completed a pre-study survey collecting demographic details, technology and care management preferences. Out of 41, 24 participants completed the study, using the app for 2 weeks and completing an exit survey. The app facilitated caregiver note-taking using voice interaction and ASR. An exit survey was conducted to collect feedback on technology adoption and changes in technology preferences in care management. We assessed the feasibility of the app by descriptively analyzing survey responses and user data following the key focus areas of acceptability, demand, implementation and integration, adaptation and expansion. In addition, perceived effectiveness of the app was assessed by comparing perceived changes in mobile app preferences among participants. In addition, the voice data, notes, and transcriptions were descriptively analyzed for understanding the feasibility of the app.

**Results:**

The majority of the recruited parents were 35–44 years old (22, 53.7%), part of a two-parent household (30, 73.2%), white (37, 90.2%), had more than one child (31, 75.6%), lived in Ohio (37, 90.2%), used mobile health apps, mobile note taking apps or calendar apps (28, 68.3%) and patient portal apps (22, 53.7%) to track symptoms and health events at home. Caregivers had experience with voice technology as well (32, 78%). Among those completed the post-study survey (in Likert Scale 1–5), ~80% of the caregivers agreed or strongly agreed that using the app would enhance their performance in completing tasks (perceived usefulness; mean = 3.4, SD = 0.8), the app is free of effort (perceived ease of use; mean = 3.2, SD = 0.9), and they would use the app in the future (behavioral intention; mean = 3.1, SD = 0.9). In total, 88 voice interactive patient notes were generated with the majority of the voice recordings being less than 20 s in length (66%). Most noted symptoms and conditions, medications, treatment and therapies, and patient behaviors. More than half of the caregivers reported that voice interaction with the app and using transcribed notes positively changed their preference of technology to use and methods for tracking symptoms and health events at home.

**Conclusions:**

Our findings suggested that voice interaction and ASR use in mobile apps are feasible and effective in keeping track of symptoms and health events at home. Future work is suggested toward using integrated and intelligent systems with voice interactions with broader populations.

## Introduction

The Maternal and Child Health Bureau defines children with special healthcare needs (CSHCN) as children “who have or are at increased risk for a chronic physical, developmental, behavioral, or emotional condition and who also require health and related services of a type or amount beyond that required by children generally” (1). Approximately 23% of households in the United States have at least one CSHCN (2). Since many care activities occur at home, outside of the clinic, caregivers (usually “unpaid caregivers” such as parents and family members) provide daily care, by tracking symptoms, medications, and health events. In addition, caregivers are frequently tasked with communicating with healthcare providers, medical suppliers, insurance companies and schools to coordinate care services. Medical diaries are often used to keep medical notes, to note symptoms and medications given, and to communicate with healthcare providers (HCP) (3). There is room for improvement for *care management* of CSHCN with digital health technologies.

The COVID-19 pandemic has increased the need for digital health technologies (DHT), especially for health management and tracking (4), remote patient monitoring (5), and telehealth (6). Investments in DHT have raised 14.7 billion USD at the first half of 2021 (7), with top funded fields focused on chronic and non-communicable conditions. In parallel, there is a growing body of literature and funded research related to digital health use in the pediatric domain (8). Studies support that available DHT (e.g., mobile phones, apps, sensors, text messages, websites) play a key role in facilitating care management and care communications (9, 10). Connected and integrated DHT (e.g., patient portals) facilitates timely communication of health activities and medical notes, reducing information barriers and improving continuous care for pediatric patients without being at the clinic (11–13). Literature further demonstrates the evidence on DHT's efficacy, feasibility, and utility in the pediatric domain (10, 14–17). However, the success of DHT in care management and communication is dependent on caregivers and patients capturing and sharing health events that occur outside of the clinic.

To improve the collection of patient notes, convenience is an important factor (18), especially for caregivers of CSCHN, who need to track care activities regularly (e.g., medications, treatment, therapies, etc.). Current digital health tools are crafted toward collecting structured patient health data and use pre-defined mechanisms to capture information (e.g., survey, checklist). Such mechanisms may be limited and miss the narratives surrounding health events (19). In addition, it can be challenging for caregivers of CSHCN to take complete notes of medical events. This is especially true at times when both their hands are devoted to providing care for their child.

To address this gap, we proposed a voice-interactive app, SpeakHealth, which enables caregivers to take medical notes through voice interaction and an automatic speech recognition (ASR) system without depending on typing or focusing on a device or screen. Voice interactive technologies (e.g., voice assistants) and ASR algorithms have been improving over the years. They enable users to command and interact with digital tools using speech and dialogue mechanisms and show promise for a variety of healthcare uses (20–23). In our earlier work (18), we prototyped the SpeakHealth app and collected feedback from parents and healthcare providers which informed the design and features of the app. In this study, we aimed to test the feasibility of the SpeakHealth app in a real world setting through a pre-post study. Participants reported their care and technology preference characteristics (pre-study), used the voice interactive app for 2-weeks to keep their medical notes, and reported their perceived care preference changes and technology adoption informed by the technology acceptance model (post-study) (24). Bowen et al.'s (25) key focus areas were used to synthesize feasibility of the app, and self-reported responses and app data was used to synthesize perceived effectiveness.

### App Components and Development Details

Building on top of the previous prototype (18), we improved user interface, features, accessibility to note taking and reviewing. The SpeakHealth app was written in JavaScript using React Native, a cross-platform development library, which accelerated development compared to writing native code for iOS (26). Data is stored, processed, and retrieved from the cloud using Amazon Web Services (AWS) (27). In particular, Cognito facilitates sign in and user creation, AppSync provides an API to perform CRUD operations on the data store, DynamoDB. S3 was used to store audio files, and lambda functions handled the transcription process. The backend was written using the AWS CDK, which describes the infrastructure as code to allow quick deployment and updating. Communication between the frontend and backend is handled using the AWS Amplify library.

### App Functionalities and Engagement

**Figure 1:**
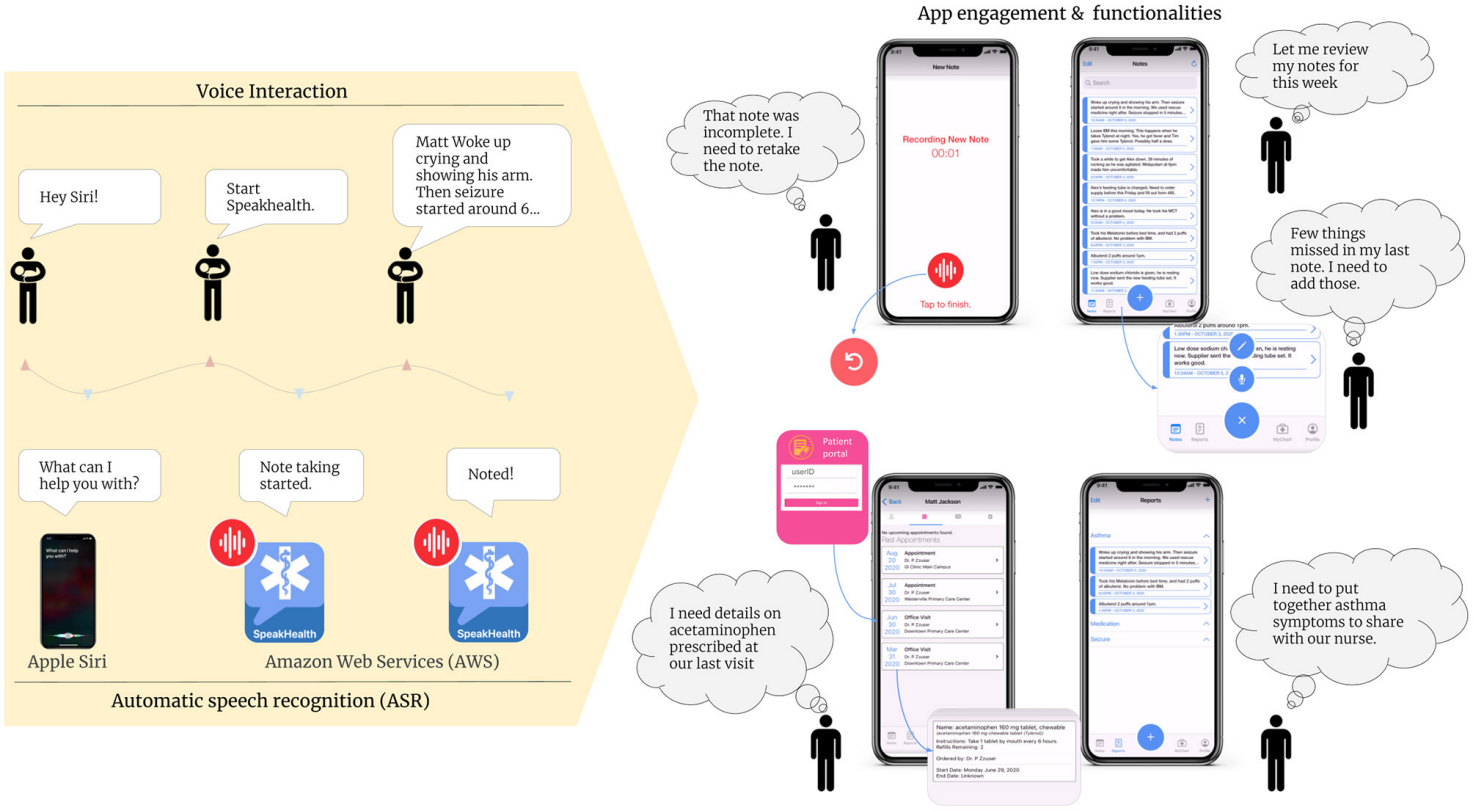
Voice interaction, ASR, and app functionalities.

Caregivers activated SpeakHealth using the Siri Shortcut “start SpeakHealth”. The app recorded the audio note until manually stopped or after the sixty-second time limit was reached (Figure 1). Then, the audio note was sent to AWS (Amazon Web Services) which performed the transcription using AWS Transcribe (28). Once the job was completed, the app updated to include the audio note's transcription. Figure 1 further outlines the app's functionalities. Caregivers were able to redo or create new audio and text notes, correct transcriptions, and delete existing notes. Caregivers could then group and categorize individual notes into reports. Reports allow caregivers to organize notes for their own benefit or to easily show grouped notes to the child's provider. Reports could also be exported as PDF for easy sharing. The app included a portal to allow caregivers to view patient medications and appointments. However, during the testing period, not all participants were able to sign into the portal due to technical errors.

## Methods

The study was designed as a pre-post study, where the SpeakHealth app was proposed as a tool for care management. Changes in participant's preferences and care management behavior were observed through pre-post surveys and data collected through the app (voice notes and transcriptions).

### Recruitment and Study Setting

Parents were invited to participate in the study through a non-probability sampling method using the network of Nationwide Children's Hospital (Columbus, OH). We sent email invitations via hospital mail-list and to the parents of children receiving care at NCH complex care clinic. The study was announced through digital boards and hospital social media channels. In addition, we worked with a local community partner (OhioF2F) to announce the study. The recruitment was time-bound, and participants were recruited between October- December 2020. Even though we are not able to access total online reach numbers, we were able to send email invites to 644 out of 1,290 patient families of children who received care at the complex care clinic in 2020.

The inclusion criteria: (1) being a parent of children who have been diagnosed with one or multiple complex medical conditions (2) being a user of an iPhone 8 (or above) or iPhone with iOS 13 or above during the study period. Forty-one participants met the inclusion criteria, consented to participate in the study and completed the introduction survey. Out of 41, 12 participants only filled out the introduction survey. Four participants filled out the introduction survey and used the app for 2 weeks. Twenty-four participants completed the full study by filling out the introduction survey, using the app for 2 weeks and submitting responses for the exit survey. Participants were compensated with a gift card up to $30 ($10 for completing the introduction survey, +$10 for participating for 2-weeks of app use, +$10 for completing the exit survey). Institutional review board of Nationwide Children's Hospital approved this study (#00000231).

### Data Collection and Analysis

Interested participants were directed to a RedCap online survey (29), where they completed eligibility screening. Eligible participants received an email providing details about the study and a link to an online consent form. Once consented, participants filled out an introduction survey, which included questions about demographics, the child's medical conditions, mobile app use and voice interactive technologies, care coordination and healthcare management, app needs and expectations (Tables 1–4 provided details on introduction survey content). Following the introduction survey, participants were guided through online tutorials for how to install and use of the app. Participants used the app for at least 2 weeks. During this period, they received periodic email reminders providing quick tips about app features (Appendix 1). Voice notes and transcriptions created through the app were collected and stored at AWS servers.

**Table 1:** Participant demographics (*n* = 41).

**Categories**	***N* (%)**
**Parent age**
18–24	1 (2.4%)
25–34	8 (19.5%)
35–44	22 (53.7%)
45+	10 (24.4%)
**Family type**
Single mother household	7 (17.1%)
Two parent household	30 (73.2%)
Single mother plus grandparent living in household	2 (4.9%)
Single mother plus extended family or friend living in household	1 (2.4%)
Other	1 (2.4%)
Single father household	0 (0)
Single father plus grandparent living in household	0 (0)
Single father plus extended family or friend living in household	0 (0)
**Race/ethnicity**
White	37 (90.2%)
Black, African American	3 (7.3%)
American Indian or Alaska Native	1 (2.4%)
**Number of children**
1	10 (24.4%)
2	15 (36.6%)
3	11 (26.8%)
4	2 (4.9%)
5+	3 (7.3%)
**Location**
Massachusetts	1 (2.5%)
West Virginia	3 (7.3%)
Ohio	37 (90.2%)
**Yearly household income before taxes**
< $20,000	4 (10.0%)
$20,000–34,999	2 (5.0%)
$35,000–49,999	5 (12.5%)
$50,000–74,999	5 (12.5%)
$75,000–99,999	11 (27.5%)
Over $100,000	13 (32.5%)
**Education**
High school degree or equivalent (e.g., GED)	3 (7.3%)
Some college, no degree	8 (19.5%)
Associate degree (e.g., AA, AS)	5 (12.2%)
Bachelor's degree (e.g., BA, BS)	15 (36.6%)
Master's degree (e.g., MA, MS, MEd)	7 (17.1%)
Doctorate (e.g., PhD, EdD)	3 (7.3%)

**Table 2:** Child conditions, treatment, and symptom tracking (*n* = 41).

**Categories**	***N* (%)**
**Child age**	
Average	7.5
Standard deviation (SD)	4.2
Range	1–17 years old
**Child's condition/diagnosis**	
Developmental delay	32 (78.0%)
Learning disability	23 (56.1%)
Speech problems	23 (56.1%)
Vision problems	23 (56.1%)
Intellectual disability	20 (48.8%)
Seizures	15 (36.6%)
Joint or muscle problems	15 (36.6%)
Cerebral palsy	15 (36.6%)
Other genetic disorders	15 (36.6%)
Epilepsy	14 (34.1%)
Asthma	11 (26.8%)
Hearing problems	10 (24.4%)
Brain injury	10 (24.4%)
Neurologic and neuromuscular disorders	10 (24.4%)
ADD/ADHD	7 (17.1%)
Behavioral problems	6 (14.6%)
Anxiety problems	5 (12.2%)
Asperger's, autism spectrum	5 (12.2%)
Down syndrome	4 (9.8%)
Diabetes	2 (4.9%)
Muscular dystrophy	1 (2.4%)
Others^*^	10 (24.4%)
**Treatments and devices being used for the child**	
Daily prescribed medications	35 (85.4%)
Physical and occupational therapy	33 (80.5%)
Behavioral or speech therapy	27 (65.9%)
Feeding tube	22 (53.7%)
Wheelchair	22 (53.7%)
Breathing assistance (BiPAP, oximeter and/or oxygen devices)	15 (36.6%)
Communication assistant device	15 (36.6%)
Suction device	14 (34.1%)
Hearing aid	7 (17.1%)
Tracheostomy	6 (14.6%)
Medical ventilation device	5 (12.2%)
Glucose Monitoring (e.g., Dexcom)	3 (7.3%)
Others^**^	10 (24.4%)
**How often do you track symptoms and health events at home in a day?**	
I track and record all symptoms and events	7 (17.1%)
I often track and record symptoms and events	22 (53.7%)
I rarely track and record symptoms and events	11 (26.8%)
I do not track and record symptoms and events	1 (2.4%)
**What kind of care activities do you track?**	
Following appointments	37 (90.2%)
Medications and refills	30 (73.2%)
Tracking symptoms	26 (63.4%)
Feeding	15 (36.6%)
Vital signs (body temperature, pulse rate, respiration rate, blood pressure)	14 (34.1%)
Behavioral activities	12 (29.3%)
Urine and/or bowel movements/ diapers	12 (29.3%)
Nursing notes	10 (24.4%)
Others^***^	4 (9.8%)

* *Cystic fibrosis, congenital heart disease, cleft lip & palate, gastrointestinal problems, DiGeorge syndrome, DDX3X syndrome, chronic lung disease, postPrandial Hyperinsulinemic hypoglycemia, scoliosis, chronic kidney disease, bronchopulmonary dysplasia, cleft lip and palate, gross motor delays, brain tumor, respiratory and swallowing problems*.

** *Vagus nerve stimulation, walk and move support, compression vest, glasses, airway clearance device and shake vest*.

*** *G-Tube replacement dates, seizure notes, seizure log, therapy progress notes*.

**Table 3:** Technology interaction in symptoms tracking symptoms, health events and care activities (*n* = 41).

**Categories**	***N* (%)**
**How do you track symptoms, health events, and care activities at home**	
**currently? (You can choose more than one)**	
Mobile health apps, mobile note taking app or calendar app	28 (68.3%)
Patient portal app (MyChart)	22 (53.7%)
Notes on paper or card	16 (39.0%)
Dedicated notebook or calendar	15 (36.6%)
Setting up reminders	13 (31.7%)
Calling/Talking to nurse	12 (29.3%)
I do not track	1 (2.4%)
Other methods^*^	4 (9.8%)
**What do you think is the ideal tool or technology to use for tracking**	
**symptoms, health events and care activities at home?**	
Mobile phone and apps	37 (90.2%)
Pen and paper/notebook	16 (39.0%)
Voice assistant (Amazon Alexa, Google Home)	13 (31.7%)
Tablet PC/iPad	10 (24.4%)
Laptop or PC	7 (17.1%)
**What is your primary source of information regarding care management?**	
**(What would you check first?)**	
Calling a nurse or doctor	25 (61.0%)
Web/internet search	24 (58.5%)
Mobile apps	12 (29.3%)
Calling a friend	3 (7.3%)
Other^**^	1 (2.4%)

* *VerbalCare, ViHealth, Nationwide Children's Hospital, CVS*.

** *Facebook groups*.

**Table 4:** Voice technology interaction.

**Categories**	***n*/*N* (%)**
**Do you interact with your smartphone with voice (e.g., using Google**	
**assistant—“Hey Google!”, Siri or Alexa app)?**	
Yes	32/41 (78.0%)
No	9/41 (22.0%)
**For how long have you been using voice interaction with your phone?**	
3–12 months	2/32 (6.3%)
1–3 years	16/32 (50.0%)
More than 3 years	14/32 (43.8%)
**Do you use voice-interactive devices/smart speakers (e.g., Amazon**	
**Alexa, Google Home, Apple HomePod)?**	
Yes	23/41 (56.1%)
No	18/41 (43.9%)
**For how long have you been using voice interaction with your**	
**smart speaker?**
3–12 months	4/23 (17.4%)
1–3 years	15/23 (65.2%)
More than 3 years	4/23 (17.4%)

At the end of this period, participants received exit surveys, which included questions about care coordination and healthcare management to assess any changes in behavior toward care activities, symptom tracking and health events after using the app. Additionally, participants responded to questions about assessing their adoption of the app. We used technology acceptance model constructs (perceived usefulness, perceived ease of use and behavioral intention) to develop this adoption questionnaire (24). Participants responded using 5-point Likert scales (0: Strongly disagree, 1: Disagree, 2: Neither agree nor disagree, 3: Agree, 4: Strongly agree).

All the data were descriptively analyzed and reported. We used descriptive analysis guided by Creswell and Creswell (30). We reported measures as frequency, mean, standard deviation, score distribution and relative ranking comparisons within the groups. The analysis was conducted separately for pre-study and post-study surveys. We used Microsoft Office tools for analysis and reporting.

## Results

### Demographics

The majority of the parents were 35–44 years old (22, 53.7%), part of a two-parent household (30, 73.2%), white (37, 90.2%), had more than one child (31, 75.6%), lived in Ohio (37, 90.2%), had a yearly household income higher than the median for the U.S. [$67.521 (31)] (29, 72.5%) and had received higher level education (undergraduate or graduate degrees) (25, 61%) (Table 1).

### CSHCN and Care Characteristics

The children with special health care needs ranged from 1 to 17 years old with an average of 7.5 years old (SD = 4.2). More than half of CSHCN had developmental delay (32, 78%), learning disability (23, 56.1%), speech problems (23, 56.1%), and vision problems (23, 56.1%). In addition, a substantial amount of CSHCN had intellectual disability, seizures, joint or muscle problems, cerebral palsy, epilepsy, asthma, hearing problems, brain injury, neurologic and neuromuscular disorders, and other genetic disorders. The majority received daily prescribed medication (35, 85.4%), physical and occupational therapy (33, 80.5%), behavioral or speech therapy (27, 65.9%), used a feeding tube (22, 53.7%) or used a wheelchair (22, 53.7%). Most of the caregivers have been tracking symptoms and health activities at home frequently (all the time or often, 29, 70.8%). These included following appointments (37, 90.2%), medications and refills (30, 73.2%), and tracking symptoms (26, 63.4%) (Table 2).

### Technology Awareness and Preferences

The majority of caregivers had been using mobile health apps, mobile note taking apps or calendar apps (28, 68.3%) and patient portal apps (22, 53.7%) to track symptoms, health events and care activities at home (Table 3). In addition, there was a preference for taking notes on a paper or card (16, 39%) or in a dedicated notebook or calendar (15, 36.6%). Mobile phones and apps had higher ratings and preference as the ideal tool or technology (37, 90.2%), followed by pen and paper or keeping notebooks (16, 39.0%), voice assistants (13, 31.7%), tablet PC or iPad (10, 24.4%), and laptop or personal computers (7, 17.1%). Care management related information seeking activities were primarily completed through nurse or doctor calls (25, 61%) and web or internet searches (24, 58.5%).

The caregivers had experience with voice technology, with the majority using voice assistants over their smartphones (32, 78%) for more than a year. In addition, almost half of them had owned voice interactive devices or smart speakers (23, 56.1%) for more than a year (Table 4).

### Post-study Analysis

#### Voice Interactive App Adoption

**Figure 2:**
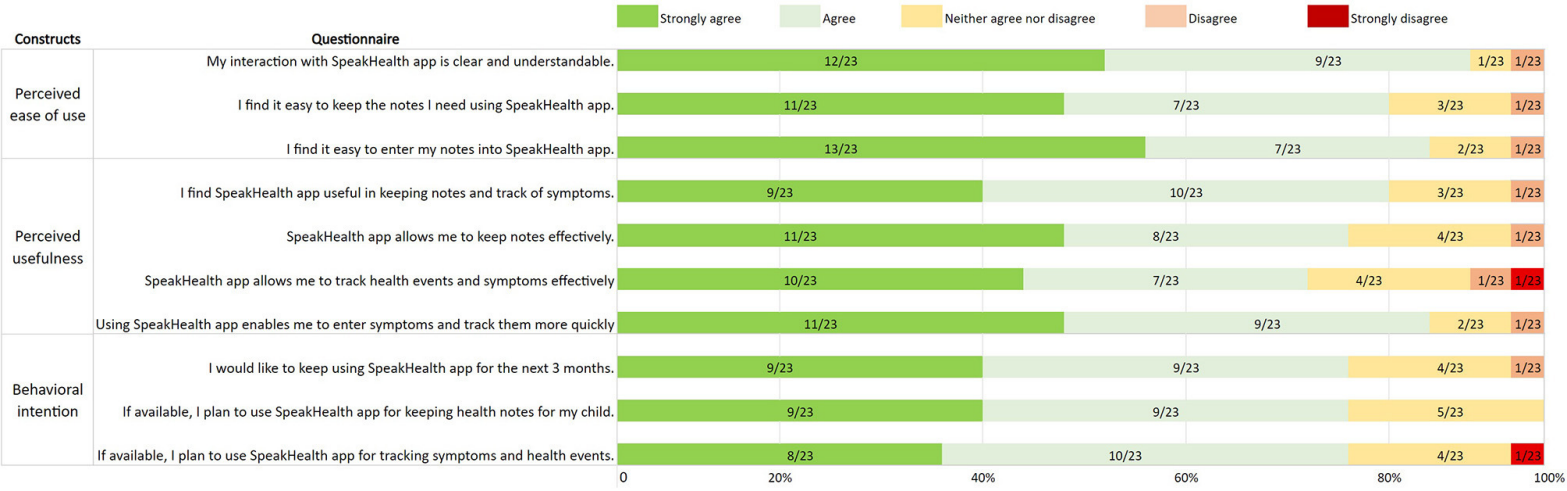
Technology adoption responses grouped under technology acceptance model constructs. Number of responses/total responses is given in each bar chart.

Feedback on the voice interactive app adoption was collected using technology acceptance model (24). Figure 2 illustrates app adoption questionnaire and response frequency distribution grouped under TAM constructs. Approximately 80% of the caregivers agreed or strongly agreed that using the SpeakHealth app would enhance their performance in completing tasks (perceived usefulness; mean = 3.4, SD = 0.8), the app is free of effort (perceived ease of use; mean = 3.2, SD = 0.9), and they would use the app in the future (behavioral intention; mean = 3.1, SD = 0.9). Given the small sample size of respondents (*n* = 23, one participant's response was removed due to highly missing data), we were not able to do statistical analysis toward explaining app use behavior. We reported the frequency distribution of responses and mean value to report user perceptions in item and construct level.

#### Characteristics of Voice Interactive Notes

In total, 95 patient notes were taken (after removing 19 “test” notes, which are created by users to try out the app). Seven out of 95 notes were taken through text entry. We had one “super-user” participant who kept 29 notes throughout the study period. Excluding that, each remaining caregiver who used the app (*n* = 23) kept 4 notes in average (SD = 2.6). Out of 88 voice interactive patient notes, most of the notes were taken in <10 s or were between 11 and 20 s in length (Table 5). Only one caregiver created note reports.

**Table 5:** Time spent on taking voice interactive notes and categories of notes.

**Time spent voice note taking**	**Frequency *N* (%)**
<10 s	19 (35.8%)
11–20 s	16 (30.2%)
21–30 s	7 (13.2%)
21–40 s	4 (7.5%)
41–50 s	2 (3.8%)
51–60 s	5 (9.4)
**Categories of notes taken**	**Frequency** ***N*** **(%)**
Symptoms/conditions	27 (18.8%)
Medication	21 (14.6%)
Treatment/therapy	20 (13.9%)
Mood/behavior	17 (11.8%)
Seizure	11 (7.6%)
Appointment	8 (5.6%)
Vital signs	8 (5.6%)
Personal notes	8 (5.6%)
Sleep	7 (4.9%)
Nutrition	6 (4.2%)
Explaining process/procedure	6 (4.2%)
Bowel movements	5 (3.5%)

The voice interactive notes were usually taken for *symptoms and conditions* (“Right shoulder pain…”, “Spot on lip is gone. Overall doing well. Has a runny nose it no fever or any other symptoms.”, “Knees hurting today…”), *medication* (“Gave [patient name] 2 Benadryl at 6:00am…”, “[patient name] does not take his medicine after lunch.”), *treatment or therapy* (“7 pm trach care completed…”), *mood or behavior* (“[patient name] was so happy that we understand him…“), *seizure* (“Starting seizure. Touched his arm no response. Eyes fixed. Lasted only a few seconds. Looked as though [patient] was staring right through me”), *appointments* (“Appointment this afternoon… mom took [patient name]”), *vital signs* (“[patient name] oxygen was still hanging out around 80 today”, “...blood sugar is 127.”), *personal notes* (“it was pointless”, “[patient] is still having a lot of pain, but we haven't had to give the strong meds again yet”, “I'm so proud of my baby”), *Sleep* (“[Patient name] still not sleeping through the night. [patient] is still waking up and crying”), *nutrition* (“Only one feed for tomorrow, 200 ml”) *explaining process/procedure* (“Yesterday we started…gabapentin at a rate of 2.6 ml that will continue for one week, then we will switch rate to 2 ml over the course of another week, then 1.6 ml for another week with final rate at 0.6 ml...”) *bowel movements* (“BM today, formed small”). A single note or entry may have multiple themes. Some notes contained a summary of the day instead of individual instances created throughout a day. For example, a caregiver may prefer to take a note about symptom, treatment, procedure, medication, and patient's mood all in one note.

Metadata included in the notes stored in AWS showed that 14 of the audio transcriptions were edited after being created, indicating errors in the transcriptions that were corrected by the participants. This was determined by comparing the timestamp of the note created against last updated and whether the note was considered a transcribed audio recording linked to an audio file. The note data was not versioned, meaning we cannot view the original transcription and how the errors were corrected. Also, since AWS Transcribe is constantly changing and improving, it is unlikely we could recreate the original transcriptions from the collected audio recordings.

#### Perceived Changes in Care Preferences

**Figure 3:**
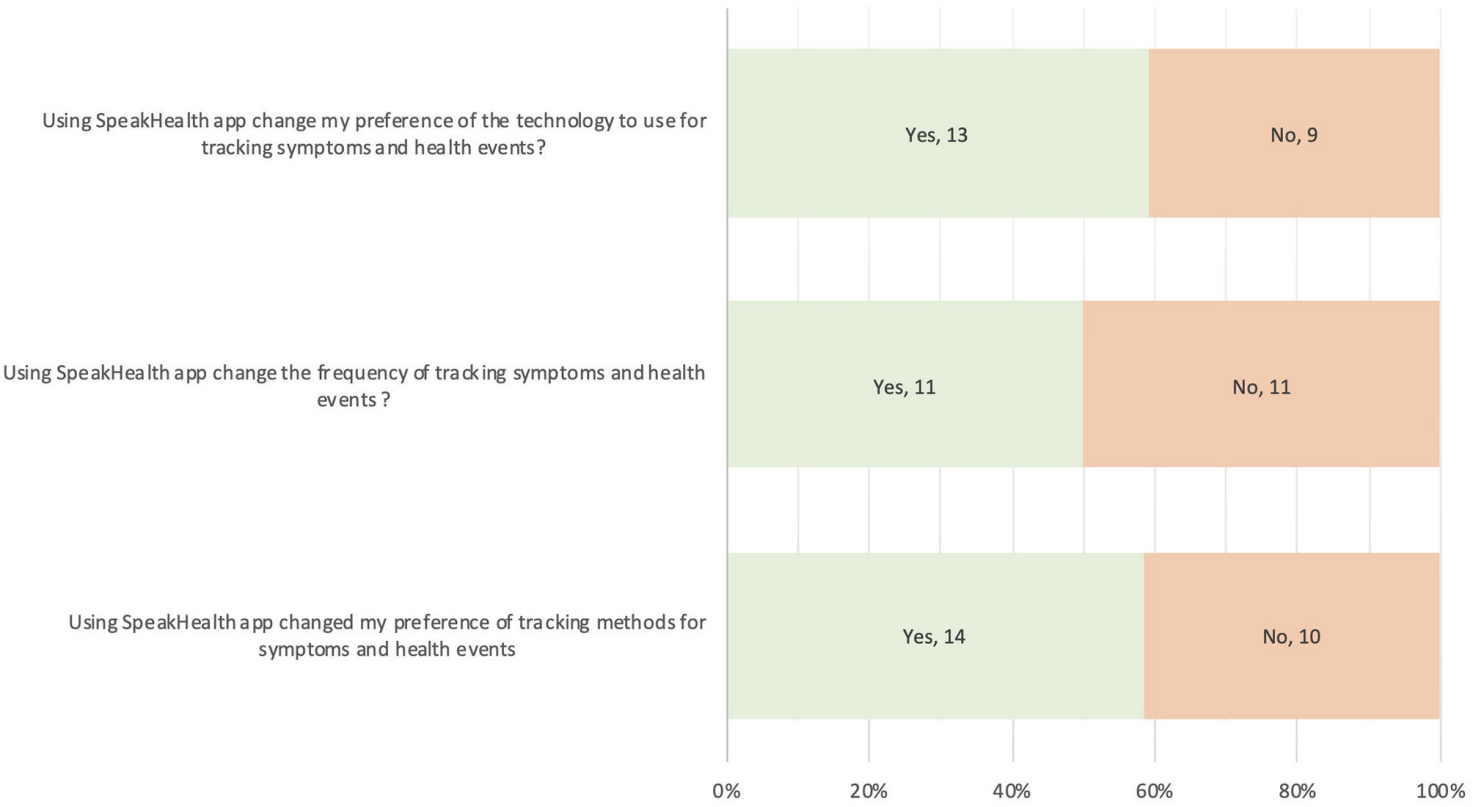
Responses to technology use preferences in care management.

Caregivers provided their feedback about their ***preference changes*** after they completed using the SpeakHealth app for at least 2 weeks (Figure 3). More than half of the caregivers reported that voice interaction with the SpeakHealth app and using transcribed notes changed their preference of technology to use (13/21, 59%) and methods for tracking symptoms and health events at home positively (14/24, 58%). Half of the caregivers changed the frequency of tracking symptoms and health events (11/22, 50%).

## Discussion

We conducted a feasibility study of a voice-enabled medical note taking (diary) app in a real world setting with caregivers (parents, family members) of children with special healthcare needs (CSHCN) to understand the impact and implications of voice interaction and automatic speech recognition for medical note taking at home. The majority of participants were young parents, with a college degree or above education, middle income level, living in a two-parent household with multiple children. Demographically, we were unable to achieve higher diversity in our data, which would have been available from a more heterogeneous group in terms of race, family type, income, and education. However, CSHCN of participants were representative, as they had various and multiple chronic conditions, and a need for frequent care management (medication, treatments, symptom tracking). The reported conditions and care management needs are in line with the national survey of CSHCN (2), the majority of which require specific care services such as medication, treatment, therapy, feeding and mobility support. Similarly, this confirms the need for care management and coordination including the ability to track health events and symptoms at home, as well as tools for easier provider communications (32, 33).

Mobile tools and technologies were the primary preference for tracking health activities and taking notes, and they were also used for health information seeking activities as part of care management. This finding aligns with the overall trend and adoption in mobile technology ownership in the U.S. (34) and caregiver preferences (13). Specifically, online patient portals have been widely used among participants. The primary reason may be that they enable direct communication with HCPs, and their wide availability through hospitals to communicate with caregivers and telehealth visits especially after the pandemic (33). This preference is supported by the common trend of using mobile apps in pediatric care management and care coordination (13, 18). Parental familiarity and use of voice assistants via smartphones and smart speakers are a promising indicator toward future utilization of voice interaction in care management. It will need to be built integrated with current healthcare technologies, moving the needle from voice interaction being primarily used for health seeking activities (21, 35) and health screening (20, 36) to the area of care management.

### Feasibility Findings

Using the data collected pre and post study, the feasibility of the app was interpreted by descriptively analyzing survey responses and user data following key focus areas adapted from Bowen et al. (25): acceptability, demand, implementation and integration, adaptation, and expansion. Perceived effectiveness was interpreted through survey responses about the perceived changes in the preferences in mobile apps used for symptom tracking and recording health activities.

#### Acceptability

In response to the voice interactive app adoption survey (*n* = 23), the majority of participants agreed that the SpeakHealth app is easy to use, useful for keeping notes and tracking health events, and that they would prefer to use SpeakHealth in the long term. Furthermore, the survey showed that SpeakHealth met parents' preferences toward the use of mobile technologies and voice interactions. Mobile apps showed similar early adoption trends among parents for care management (37). As voice technologies become more aligned with current habits and lifestyles, increased adoption of the technology can be expected.

User data for voice interactive notes showed that caregivers are interested in voice engagement and note taking for shorter notes most of the time (<20 s), and toward noting symptoms and conditions, medications, treatment and therapies, and patient behaviors. Shorter notes principally increase effective note taking, and potentially accuracy in transcriptions since shorter sentences are often less complex. Voice engagement may change note taking behavior over time, especially when integrated with other smart devices (e.g., smart speakers). Multimodal voice interactive technologies should be implemented in the future to assess acceptability using multiple platforms and digital ecosystems.

#### Demand

The overall demand for mobile apps and voice technologies is increasing. As of today, 97% of the U.S. adult population have a mobile device (34) and 35% own smart speakers (38). This suggests potential infrastructure availability, as well as general awareness and demand for these communication tools. In our study, even though we did not have the equal race distribution, we had participants from low income (*n* = 6) and limited education populations (no degree or high school degree, *n* = 11), yet overall feedback toward using mobile and voice interactive apps was positive. Similar to the literature, there was a demand on mobile technology to improve health outcomes (39, 40). The responses to the adoption survey and the collected voice interactive notes show the distribution of technology and voice interaction across demographics. However, for further implementations, digital equity, access, and digital literacy should be assessed to improve engagement of voice interactive apps.

#### Implementation and Integration

The implementation of voice engagement through SpeakHealth was convenient, since caregivers were able to use the app on their own phone and to access the note taking services. SpeakHealth was low cost to develop and nested in the integrated ecosystem of the mobile phone, allowing users to install and use the app without needing any added instruction. Also, the ratio between voice interactive notes taken and notes being edited was low, indicating AWS ASR was able to capture the intended text most of the time. Overall, mobile platforms and ASR technology showed competence in our study. Even so, we do not have the knowledge of what errors led to participants editing transcriptions during the study. Potentially they could be related to complex sentences or medical terms or interference from noisy environments (41). Should that be the case, such occurrences may potentially lead to frustration over time.

#### Adaptation and Expansion

In our study, we provided a glimpse of how speech technologies can be utilized in care management for caregivers with CSHCN. Large scale adaptation and expansion of the voice interactive care management tools is possible. As Amazon, Google and Microsoft provide more HIPAA compliant AI-as-a-services (which range from ASR, NLP models, speech-to-text, text-to-speech, and translation mechanisms) modern cloud and mobile application ecosystems will become increasingly easier to integrate into patient-facing apps at scale (with minimal quality of service loss for large user bases and populations). Such services are constantly improving, and the accuracy can be expected to increase over time (e.g., Amazon Medical Scribe for medical terms in transcriptions) (42).

#### Perceived Effectiveness

After using SpeakHealth, caregivers shared their feedback on their perceived changes toward technology use, frequency and methods in tracking symptoms and health events. They displayed a preference to use voice interactive apps for note taking activities and increase in perceived effectiveness in care management. Even though it is limited observation, the change is promising that voice interactive apps may increase the frequency of tracking symptoms over time. Effectiveness of voice interaction has already been demonstrated in daily use (43, 44). However, our observation should be further investigated with a larger sample size (and data points) in a longitudinal study.

The convenience of using natural language instead of a screen gives caregivers the ability to take ongoing notes on care management while simultaneously providing home care. It allows for better family engagement and less distraction, especially when a caregivers' attention is primarily directed toward caring for a child. Voice assistant-based apps have already been implemented for care management among the adult and elder populations with chronic conditions (45). In the future, SpeakHealth-like mechanisms could be integrated with smart devices featuring ambient implementation with voice assistants to be a valuable partner in care management (46), building early warning mechanisms for personalized health (19) and public health (20), contributing to personal medical notes as supporting the shared decision making (47), medication adherence and treatment (48) and improving patient-reported outcomes with integration into the electronic medical records (49).

The use of “defaults” in technology (Siri as default voice assistant) and human nature (speech as default communication mechanism) creates a window of opportunity to improve human-computer engagement (22, 50, 51). A voice user interface (VUI) with applications helps to overcome barriers of language, literacy and improve digital equity and telehealth practices. Interoperable and integrated systems with hospital EHR may also improve care coordination, and eventually reduce hospital admissions (52). Health outcomes could be informed by the rich unstructured patient-generated health data (PGHD) (19) and speech biomarkers collected through the audio notes (53). Yet, practitioners should be aware of potential infrastructure, legal and practical barriers implementing voice interactive healthcare practices (20, 21, 54, 55).

### Strengths and Limitations

The strength of our study was that it provided a new perspective about the cohort of caregivers of CSHCN, by investigating their preferences, perceptions and engagement using one of the emerging technologies in health care. The results can present an understanding of the responses of participants to create outcome measures in the future.

In our study, we had several limitations. The recruitment was online, therefore verifying parents meeting inclusion criteria was limited. We were not able to gather the total number of reaches as social media, therefore the response rate is unknown. We had a high number of dropouts from the pre-survey group, which limited the analysis. We did not conduct sample size calculations, but this study will help to estimate the effective sample size for future larger scale studies. We were not able to investigate association between voice notes, condition, and demographics due to limited sample size and voice notes taken through statistical analysis. Similarly, we were not able to measure health outcomes or comparative effectiveness of the app use (vs. currently used methods) through standardized measures, and therefore, only able to report perceived effectiveness through the post-study survey. In addition, the demographic distribution skewed toward white race, higher education received, middle income, younger and technology user parents. More input from marginalized or underserved groups regarding the use of voice interactive technologies is needed, as the results could be impacted by the performance of ASR (56). The study duration was limited (2-weeks) to achieve long term observation toward behavior change in note taking and assess the impact of the app. Finally, our study did not capture differences between entered text and transcribed notes.

## Conclusions

We reported a pre-post study for assessing feasibility of voice interaction and ASR via a mobile app (SpeakHealth). This implementation mimicked the interaction and a real-world use of mainstream voice assistants on smart devices. Our findings suggest that voice interaction and ASR use in mobile apps are feasible and effective in keeping track of symptoms and health events at home. Future work is suggested toward integrated and intelligent systems with broader populations.

## Data Availability Statement

The datasets presented in this article are not publicly available due to the inclusion of personal health information (PHI). Requests to access the datasets should be directed to emre.sezgin@nationwidechildrens.org.

## Ethics Statement

The studies involving human participants were reviewed and approved by Institutional review board of Nationwide Children's Hospital. The patients/participants provided their written informed consent to participate in this study.

## Author Contributions

ES, YH, and GN conceived of the presented idea. ES developed the research design, conducted the study, and drafted the manuscript. GN assisted the recruitment. YH and GN supported and supervised the study. BO developed the app. BA supported the app design. All authors discussed the results and contributed to the final manuscript.

## Funding

This study was supported by the Health Resources and Services Administration Maternal and Child Health Bureau Grand Challenge for Care Coordination for CSHCN (# 720467022000).

## Conflict of Interest

The authors declare that the research was conducted in the absence of any commercial or financial relationships that could be construed as a potential conflict of interest.

## Publisher's Note

All claims expressed in this article are solely those of the authors and do not necessarily represent those of their affiliated organizations, or those of the publisher, the editors and the reviewers. Any product that may be evaluated in this article, or claim that may be made by its manufacturer, is not guaranteed or endorsed by the publisher.
