# Supplementary material for: “Hey Siri, Help Me Take Care of My Child”: A Feasibility Study With Caregivers of Children With Special Healthcare Needs Using Voice Interaction and Automatic Speech Recognition in Remote Care Management
Source: Front Public Health. 2022 Mar 3;10:849322. doi: 10.3389/fpubh.2022.849322 (PMC8927637; doi:10.3389/fpubh.2022.849322)
Supplement: Supplementary file 1 [file Table_1.DOCX]

# Appendix 1. Reminders to use over email

| **Days after study start date** | **subject of the message** | **Message content** |
| --- | --- | --- |
| 0 | Welcome to SpeakHealth study | Brief introduction of the app and how to use guideline |
| 3 | More to do with SpeakHealth app | Reminder to take notes and quick tips on taking notes through voice or text |
| 6 | More to do with SpeakHealth app | Reminder to take notes and quick tips about editing notes |
| 9 | More to do with SpeakHealth app | Reminder to take notes and quick tips about deleting notes and creating reports by combining notes |
| 12 | Getting closer to the end- SpeakHealth study | Reminder to take notes |
| 14 | SpeakHealth study is completed | Exit survey and compensation details |
| 16 | SpeakHealth study is completed - what is next? | Reminder for exit survey and compensation details |
